# A prospective cohort study of ultra-rush subcutaneous immunotherapy in dust mite-induced allergic rhinitis^☆^

**DOI:** 10.1016/j.waojou.2026.101334

**Published:** 2026-02-06

**Authors:** Yishan Xiong, Wenqi Luo, Hao Peng, Li Shen, Zhiqun Huang, Jieqing Yu, Jing Ye

**Affiliations:** a Department of Otorhinolaryngology-Head and Neck Surgery, The First Affiliated Hospital, Jiangxi Medical College, Nanchang University, Nanchang, Jiangxi, PR China; b Jiangxi Medicine Academy of Nutrition and Health Management, Nanchang, Jiangxi, PR China; c Allergy Department, The First Affiliated Hospital of Nanchang University, Nanchang, Jiangxi, PR China

**Keywords:** Desensitization, Immunologic, Ultra-rush protocol, Rhinitis, Allergic, Dust mites, Adverse reactions

## Abstract

**Background:**

Allergic rhinitis (AR) is a prevalent disease with a considerable global burden. Allergen immunotherapy (AIT) represents the cornerstone causal treatment for AR. While accelerated schedules such as Cluster Subsutaneous Immunotherapy (Cluster SCIT) shorten the initial buildup phase than Conventional treatment, the even more rapid Ultra-Rush Subcutaneous Immunotherapy (UR-SCIT) lacks a head-to-head, prospective comparison regarding its efficacy and safety.

**Methods:**

A total of 57 patients with house dust mite induced AR were included in this study and followed up for 12 months. Allocation to the 2 treatment groups (UR-SCIT and Cluster SCIT) was based on patient preference. Treatment efficacy was assessed using Visual Analogue Scale (VAS), Rhinoconjunctivitis Quality of Life Questionnaire (RQLQ) and Combined Symptom and Medication Score (CSMS). Additionally, skin prick test (SPT), nasal allergen challenge (NAC), fractional exhaled nitric oxide (FeNO), and fractional nasal nitric oxide (FnNO) were measured at baseline (M0) and at the 12-month follow-up (M12). Eosinophils (EOS) and eosinophil percentage (EOS%) in blood、serum levels of IL-4, IL-5, IL-10, IL-13, IL-33, total IgE, dust mite-specific IgE (sIgE), dust mite-specific IgG4 (sIgG4),the ratio of sIgG4/sIgE and sIgE/tIgE were detected at months 0, 6, and 12. To evaluate safety, local and systemic adverse reactions were recorded during the treatment period.

**Results:**

In this study, both UR-SCIT and Cluster SCIT groups showed significant reductions in VAS, CSMS, RQLQ and TNSS scores at M12, alongside decreases in SPT grade. Immunological analysis revealed that both groups exhibited reduced levels of EOS, EOS%, IL-4, IL-5, IL-13 and IL-33, while IL-10 levels increased. Changes in EOS count and EOS% levels were correlated with improvements in AR symptoms. SIgG4 levels rose in both groups, but tIgE、sIgE and sIgE/tIgE ratios showed no significant changes. Adverse reaction incidence was similar in both groups, with no serious events reported.

**Conclusion:**

UR-SCIT achieves therapeutic efficacy comparable to Cluster SCIT, with both demonstrating robust symptom control and favorable immunological changes. The similar safety profiles, with no serious adverse events indicate that UR-SCIT is a viable new option for AIT in clinical practice.

## Introduction

Allergic rhinitis (AR) is a chronic type I hypersensitivity disorder of the nasal mucosa mediated by immunoglobulin E (IgE) in atopic individuals, characterized by paroxysmal and recurrent symptoms such as sneezing, watery rhinorrhea, nasal itching, and congestion due to inhalant allergens.^1^, ^2^, ^3^ As a globally prevalent chronic respiratory disease, AR affects 5%–50% of the population, with 10%–30% of adults and up to 40% of children.^4^ Furthermore, AR is associated with comorbidities such as bronchial asthma, sinusitis, nasal polyps, and secretory otitis media. Current therapeutic strategies for AR encompass environmental control, pharmacotherapy, immunotherapy, and health management.^5^^,^^6^ Allergen-specific immunotherapy (AIT) achieves sustained clinical remission by gradually exposing patients to the standardized allergen extracts, thereby inducing antigen-specific immune tolerance, mitigating hypersensitivity, and promoting long-term alleviation of allergy-related symptoms.^7^^,^^8^

Subcutaneous immunotherapy (SCIT), a cornerstone of AIT, achieves allergen-specific immune desensitization through initial build-up and maintenance phases. Based on dose-escalation protocols, SCIT is categorized into conventional immunotherapy and accelerated immunotherapy.^9^^,^^10^ The latter is further classified into Cluster SCIT, rush immunotherapy (Rush SCIT), and ultra-rush SCIT (UR-SCIT). Both Cluster and Rush SCIT have been widely applied in the world, and their safety profiles have been validated.^11^^,^^12^ The initial phase of Cluster SCIT is typically completed within 6–10 weeks, requiring once-weekly clinic visits for subcutaneous injections. However, the lengthy initial schedule of Cluster SCIT imposes substantial practical burdens on patients, including increased travel expenses and time commitments. In contrast, Rush SCIT reach the maintenance dose within a significantly shorter duration, generally in 1 week, through an accelerated injection regimen. Rush SCIT has been employed in several domestic institutions in China, without serious adverse reactions (SARs) have been reported to date.^13^, ^14^, ^15^

UR-SCIT involves administering multiple escalating doses of allergen extracts over 1–2 days, followed by transition to the maintenance phase. Reviews of the past decade's research indicate that UR-SCIT has been primarily applied for hymenoptera/insect venom and mixtures of aeroallergen.^16^^,^^17^ Crucially, no prospective cohort trials have directly compared UR-SCIT and Cluster SCIT for house dust mite (HDM) AR, creating a critical evidence gap for clinical adoption.

## Materials and methods

### Clinical data

This prospective cohort study evaluated the safety and efficacy of UR-SCIT in patients with perennial AR primarily sensitized to HDMs. This study is registered with the Chinese Clinical Trial Registry (prospectively registered; Date: June 23, 2022). The protocol also received approval from the local Institutional Review Board, dated June 2, 2022. All participants provided written informed consent prior to enrollment. The study was conducted in accordance with the Declaration of Helsinki and ethical standards for clinical research.

### Patients information

Patients were recruited from the outpatient department, eligible patients were comprehensively informed about both UR-SCIT and Cluster SCIT protocols, including their respective efficacy, safety profiles, and the risk of systemic reactions (SRs) during UR-SCIT using HDM extracts and provided written informed consent. Subsequently, patients were allocated to their preferred treatment group. Inclusion criteria followed the Allergic Rhinitis and its Impact on Asthma (ARIA) guidelines.^18^ Patients aged 14–65 years with dust mite-induced Perennial AR. Diagnostic criteria included: (a) persistent nasal symptoms such as rhinorrhea, sneezing, and nasal congestion, and (b) Patients were diagnosed based on either a positive skin prick test (SPT) or a serum specific IgE (sIgE) level ≥ grade 2. Sensitization to other allergens (eg, pollen or food) was permitted, provided that patients had no history of significant symptomatic rhinitis upon exposure to these allergens. (The profile of co-sensitizations is summarized in Table 1). SIgE values were categorized into 7 grades: sIgE <0.35 kU/l, grade 0 (negative); 0.35–0.7 kU/l, grade 1; 0.7–3.5 kU/l, grade 2; 3.5–17.5 kU/l, grade 3; 17.5–50 kU/l, grade 4; 50–100 kU/l, grade 5; sIgE ≥100 kU/l, grade 6.

**Table 1:** The characteristics and laboratory findings at baseline of the study population

Characteristic	UR-SCIT(*N* = 32)	Cluster SCIT(*N* = 25)	*P* value
Age (years), median (IQR)	23 (19–33)	33 (24–37)	0.053
Male/Female	13/19	11/14	0.832
Smoking history, yes:no	5/27	3/22	0.74
VAS average score, mean, 95% CI	2.95 (2.56–3.35)	3.34 (2.64–4.03)	0.298
RQLQ average score, mean, 95% CI	2.35 (2.09–2.62)	2.58 (2.13–3.03)	0.659
CSMS average score, mean, 95% CI	3.13 (2.81–3.44)	3.28 (2.95–3.61)	0.906
NAC			0.760
1:100	8	5	
1:1000	24	20	
FeNO (ppb) mean, 95% CI	37.91 (27.37–48.45)	30.24 (23.57–36.91)	0.667
FnNO (ppb) mean, 95% CI	516.8 (446.6–587.1)	567 (437.9–696.2)	0.822
EOS count, median (IQR)	0.29 (0.14–0.44)	0.22 (0.11–0.44)	0.353
EOS%, median (IQR)	3.9 (2.05–6.45)	2.65 (1.64–5.8)	0.489
Total IgE (IU/ml), median (IQR)	189 (78.25–361.5)	174.6 (70.1–300.50)	0.761
sIgE (IU/ml), median (IQR)	9.17 (7.43–18.23)	26.52 (20.49–48.71)	0.004
IL-4, median (IQR)	4.10 (2.28–7.55)	6.491(4.45–11.91)	0.099
IL-5, median (IQR)	5.06 (3.28–9.1)	5.31 (2.87–7.93)	0.649
IL-10, median (IQR)	7.85 (2.475–15.39)	11.23 (6.29–37.12)	0.051
IL-13, median (IQR)	4.92 (2.29–8.26)	6.69 (2.58–10.07)	0.533
IL-33, median (IQR)	76.41 (33.02–108.3)	52.03 (27.65–69.18)	0.072
sIgG4, median (IQR)	92.27 (67.18–170)	91.97 (23.45–156.3)	0.436
Comorbid other allergens	8	7	0.999
Pollen allergy	4	3	
Drug allergy	1	2	
Food allergy	3	2	

**Abbreviations:** IQR, interquartile range; VAS, Visual Analog Scale; RQLQ, Rhinoconjunctivitis Quality of Life Questionnaire; CSMS, Combined Symptom and Medication Score; NAC, nasal allergen challenge; FeNO, fractional exhaled nitric oxide; FnNO, fractional nasal nitric oxide; EOS, eosinophils; IL, interleukin; sIgG4, specific immunoglobulin G4. **Note:** Sensitizations to other allergens are listed for completeness; house dust mite was the sole clinically relevant allergen for all enrolled patients based on their clinical history.

Exclusion criteria covered several categories: current use of β-blockers or angiotensin-convertising enzyme inhibitors (ACEIs); significant comorbidities in major organ systems, including uncontrolled hypertension or complicated/long-standing diabetes; a history of asthma or relevant ear nose and throat (ENT) pathologies (eg, nasal polyps, sinusitis); recent participation in other trials or immunotherapy; use of immunosuppressive drugs; history of severe allergic reactions (SARs), angioedema, or malignancy; pregnancy, breastfeeding, or planned pregnancy within 1 year; and active substance abuse or psychiatric conditions that could impair the ability to provide informed consent. The study cohort comprised 57 allergic rhinitis patients (age ≥14 years, range 14–60) with dust mite as the primary sensitizing allergen, meeting inclusion criteria. Pre-treatment pulmonary function tests were performed to exclude participants with underlying asthma.

### Clinical questionnaires

Visual analog scale (VAS): A 10-cm horizontal scale (0 = “best”, 10 = “worst”) for patients to rate treatment efficacy over the past week. A reduction in the VAS score by ≥ 2 points from baseline or a relative reduction of ≥30% is considered indicative of effective treatment; otherwise, the treatment is deemed ineffective. Rhinoconjunctivitis Quality of Life Questionnaire (RQLQ): assessed AR impact on quality of life via 28 items. Combined Symptom and Medication Score (CSMS): a comprehensive evaluation of nasal symptoms and medication use. Oral and/or topical (ocular or nasal) use of non-sedating H1 antihistamines is scored as 1 point, use of nasal corticosteroids (regardless of whether they are combined with H1 antihistamines) is scored as 2 points, and oral corticosteroids (regardless of whether they are combined with H1 antihistamines and nasal corticosteroids) are scored as 3 points. The total score ranges from 0 to 6. A relative reduction of ≥30% signifies effective treatment, whereas a ΔCSMS value below 30% implies ineffective treatment. Total Nasal Symptom Score (TNSS): evaluated nasal itching, congestion, sneezing, and rhinorrhea (0–3 points per symptom)with a total score of 12.

### Skin prick test (SPT)

SPT was performed using histamine (positive control) and standardized allergen extracts (ALK-Abelló, Hørsholm, Denmark). SPT-positive reactions were graded using the skin index (SI), with SI representing the ratio of the mean diameter of allergen wheals to the mean diameter of histamine wheals, categorized as follows: negative (SI < 0.3), class 1 (SI = 0.3 to 0.5), class 2 (SI 0.5 to 1.0), class 3 (SI 1.0 to 2.0), or class 4 (SI ≥ 2.0).

### Nasal allergen challenge (NAC)

Using standardized freeze-dried extracts of house dust mites (*Dermatophagoides pteronysinus*) (eg, ALK Abelló, Denmark), dissolved in diluent (Aquagen SQ, ALK) at a concentration of 1000 SBU/ml. Symptoms were evaluated on a four-point scale: 0 (no symptoms) to 3 (severe symptoms) for sneezing, nasal discharge, itching, and nasal obstruction (Total score range 0–12). Total nasal symptom scores were recorded at screening, before challenge and at 10 min after each NAC. The effect of treatment was assessed by average TNSS score.

### Procedure and premedication

All patients received standardized house dust mite allergen extracts (ALK-Abelló, Hørsholm, Denmark) for AIT. The allergen extracts were divided into 4 vials with progressively increasing concentrations: 100, 1000, 10,000, and 100,000 SQ-U/mL. UR-SCIT consisted of incremental doses from vials 1, 2, 3, and 4 on days 1–3, followed by a maintenance dose (100,000 SQ-U) on day 4 and week 5. Cluster SCIT included a 6-week dose-escalation phase, reaching 100,000 SQ-U/mL by week 7. A summary of the dosing schedules is provided in Table 2, and a schematic overview is presented in Fig. 1A. Arrows indicate subcutaneous injections, with therapeutic efficacy evaluated at baseline (M0), month 6 (M6), and month 12 (M12). At these time points, clinical questionnaires were administered, and blood samples were collected for immunological analysis (Fig. 1A).

**Table 2:** Detailed dose-escalation schedules for UR-SCIT and Cluster SCIT

UR-SCIT				
Visit (Week.Day)	Vial no.	Volume (ml)	Dose (SQ-U)	Time
W1D1^a^	1	0.1	10	9:00
	2	0.1	100	10:00
	3	0.1	1000	11:00
	3	0.3	3000	13:00
	3	0.5	5000	14:00
	3	1.0	10,000	15:00
	4	0.1	10,000	16:00
W1D2^a^	4	0.1	10,000	9:00
	4	0.2	20,000	10:00
	4	0.4	40,000	11:00
W1D3^a^	4	0.5	50,000	9:00
	4	0.5	50,000	11:00
W1D4^a^	4	1.0	100,000	9:00
W5	4	1.0	100,000	9:00
One maintenance dose injection every 4–6 weeks …...				

Cluster SCIT(continued)				
Visit (Week)	Vial no.	Volume (ml)	Dose (SQ-U)	Time
W1	1	0.2	20	9:00
	1	1.0	100	9:30
	2	1.0	1000	10:00
W2	3	0.2	2000	9:00
	3	0.4	4000	9:30
W3	3	0.6	6000	9:00
	4	1.0	10,000	9:30
W4	4	0.1	10,000	9:00
	4	0.2	20,000	9:30
W5	4	0.2	20,000	9:00
	4	0.4	40,000	9:30
W6	4	0.4	40,000	9:00
	4	0.6	60,000	9:30
W7	4	1.0	100,000	9:00
One maintenance dose injection every 4–6 weeks				

**Abbreviations:** SCIT, subcutaneous immunotherapy; SQ-U, standardized quality units.

a Premedication (32 mg methylprednisolone and 10 mg loratadine) was administered before injections at visits.

**Fig. 1:**
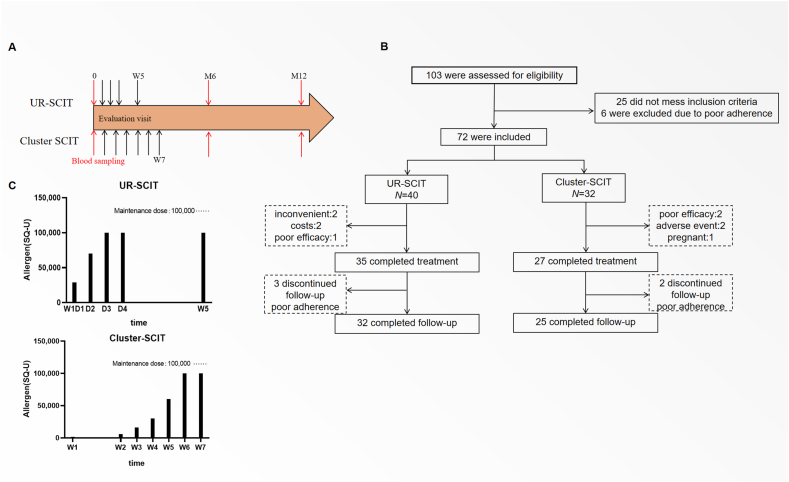
**Patient enrolment and study design.** (A) Schematic timeline of the ultra-rush (UR) SCIT (upper panel) and Cluster SCIT (lower panel) protocols. Black arrows indicate subcutaneous injections; Red arrows denote the time points for efficacy evaluations (M0, M6, M12). (B) Patient enrollment and follow-up flowchart. (C) Cumulative dose (SQ-U) received during the build-up and maintenance phases of UR-SCIT versus Cluster SCIT

To mitigate the risks associated with rapid dose escalation, a premedication regimen of oral corticosteroids (32 mg) and H1-antihistamines (loratadine, 10 mg) was administered. Premedication with oral corticosteroids (32 mg) and loratadine (10 mg) was administered once daily on Days 1–4 of the initial buildup phase to the UR-SCIT group. The Cluster SCIT group did not receive premedication (Table 2).

### Safety monitoring

Peak expiratory flow (PEF) was measured pre-and post-injection and the average value was calculated. If the average PEF fell below 80% of the predicted value, treatment was resumed only after symptoms were alleviated; if it dropped below 70%, the injection was paused. Monitor patients for at least 30 min after injection. Protocol details were summarized in Table 2.

### Blood eosinophils (EOS)

Changes in blood eosinophils counts and percentages were evaluated before treatment and at M6 and M12.

### Immunological testing

Serum was separated by centrifugation (3000 rpm for 10 min), and Serum was preserved at −80 °C. Total IgE (tIgE) and dust mite-specific IgE (sIgE) levels(IU/ml) were quantitatively measured using the Sharay 4000 fully automated chemiluminescence analyzer equipped with magnetic particle-based chemiluminescence immunoassay and inhaled allergen-specific reagent kits. Dust mite-specific IgG4 was quantified using a sandwich ELISA kit (Shanghai Baili Biotechnology, China). The levels of interleukins (IL-4, IL-5, IL-10, IL-13, IL-33) were determined using standardized assay kits (Boster Biological Technology, Wuhan, China). Absorbance was measured using a multimode microplate reader (Thermo Fisher, USA).

### Adverse reactions assessment

Table 3 summarizes the incidence, severity, type, and timing of systemic reactions. Local reactions included erythema, swelling, or pruritus at the injection site. Systemic reactions (eg, urticaria, wheezing, pharyngitis) were graded per the WAO AIT Systemic Reaction Grading System. Grades 1 and 2 are designated as mild and moderate systemic reaction events, and grades 3 to 5 are designated as severe anaphylaxis events.^19^ Local reactions were managed with ice or antiallergic drugs. In case of grades 1–2 systemic adverse reactions: fractionated injection with prolonged intervals or a dose reduction to the previous injection level, along with symptomatic and supportive treatment. Administer a block using 0.1–0.2 ml of epinephrine solution (1:1000), establish IV access, give antihistamines such as diphenhydramine and rapid-acting β2-agonists, and administer IV aminophylline. Grade 3–5 (Severe/Anaphylaxis), involving laryngeal edema, hypoxia (SpO2 < 92%), or refractory hypotension, mandated repeat IM epinephrine every 5–15 min, aggressive fluid resuscitation, and permanent discontinuation of UR-SCIT in cases of life-threatening reactions. For analysis, the incidence and severity of SRs were evaluated on both a per-patient and a per-injection basis (Table 3).

**Table 3:** Characteristics of systemic reaction (SR) during the initial buildup phase of UR-SCIT and Cluster SCIT

Characteristic	UR-SCIT	Cluster SCIT	χ2 value/Fisher test	*P* value
Number of patients	32	25		
**Patients with SR(%)**	7 (21.9)	5 (20)	0.045	0.832
Intensity				
Mild(%)	4 (12.5)	3 (12)		
Moderate(%)	3 (9.4)	2 (8)		
Severe(%)	0 (0)	0 (0)		
Number of injection times	800	625		
**Per-injection analysis**				
Injections with SR, n (%)	32 (4.0)	24 (3.84)	0.024	0.876
Intensity				
Mild(%)	22(2.75)	19(3.04)		
Moderate(%)	10(1.25)	5(0.8)		
Severe(%)	0	0		
**Timing of SRs**				0.99
Occurred during initial 4-day buildup (UR-SCIT)/7-week buildup (Cluster SCIT),n (%)	27(84.4)	18 (75)		

**Abbreviations: SR,** systemic reaction; SR were graded per the WAO AIT Systemic Reaction Grading System.

### Blinding

Because of the intrinsic characteristics of the 2 immunotherapy regimens, complete blinding of participants and treating clinicians was not feasible. To minimise assessment bias, all study personnel responsible for eliciting, recording, and managing patient-reported outcomes were kept unaware of treatment allocation. In addition, the statisticians remained blinded to group identity until analyses were complete.

### Statistical analysis

Data were analyzed using SPSS 26.0, and an *a priori* power analysis for the primary safety endpoint (adverse allergic reaction rate) was conducted with PASS 15.0 (α = 0.05, power = 0.80). Normality assessed by the Shapiro-Wilk test. Between-group comparisons for continuous variables were performed using independent samples t-tests or Mann-Whitney U tests, as appropriate. Categorical variables were compared using the Chi-square test or Fisher's exact test. Effect sizes for continuous outcomes are reported as mean differences with 95% confidence intervals. Longitudinal efficacy data were analyzed using repeated-measures ANOVA. To handle missing data, a sensitivity analysis was performed using multiple imputation on the Full Analysis Set (FAS; *N* = 72), which included all patients receiving at least 1 dose. The primary efficacy analysis was based on the Completer Analysis Set (CAS; *N* = 57). Categorical data were analyzed using the χ2 test or the Kruskal-Wallis rank sum test, depending on whether they were unordered or ordered. Adverse reaction rates were analyzed per patient and per injection using Fisher's exact test. All statistical tests were two-tailed, with significance set at *P* < 0.05.

## Results

### Clinical demographics

From June 2022 to December 2023, 72 patients met the inclusion criteria, with 57 patients achieving 12-month follow-up. The cohort comprised 32 participants in the UR-SCIT group and 25 in the Cluster SCIT group. In the UR-SCIT group, 2 patients withdrew due to transportation inconvenience, 2 due to high treatment costs, 1 due to poor treatment response, and 3 were lost to follow-up by the year. In the Cluster group, 2 patients discontinued due to poor efficacy, 2 due to adverse reactions (asthma, wheezing, chest tightness), and 1 due to pregnancy, 2 were lost to follow-up (Fig. 1B). The UR-SCIT regimen achieved the maintenance dose in a rapid, four-day escalation, whereas the Cluster SCIT regimen required a 7-week, once-weekly schedule to reach the same dose. The detailed escalation steps for both protocols are outlined in Fig. 1C. Baseline data, including gender, age, smoking history, allergy history, symptom scores (VAS, RQLQ, CSMS), SPT, FeNO/FnNO, blood eosinophil count, total IgE were comparable between groups (Table 1). UR-SCIT group presented significantly lower median sIgE than Cluster SCIT group. To exclude confounding, we re-analyzed primary efficacy end-points (change of VAS score from baseline to month 12) with ANCOVA. After adjustment,the between-group differences in symptom improvement remained statistically non-significant (eg, for VAS: adjusted mean difference 0.6, 95%CI −0.12 to 1.4, *P* = 0.09; both RQLQ and CSMS, *P* > 0.05). These results confirm that the observed baseline imbalance in sIgE did not materially influence the primary efficacy conclusions of the study.

### Clinical efficacy

At baseline, no significant differences were observed in the VAS, RQLQ, or CSMS scores between the 2 groups. The Cluster group showed reduced trends on the VAS, RQLQ, and CSMS scores at Month 6 and 12 (*P <* 0.05) (Fig. 2A–C). UR-SCIT achieved comparable VAS reduction to Cluster SCIT at M12 (Mean difference = −0.130, 95% CI: −1.364 to 1.105, *P* > 0.99), though early improvement at M6 was less pronounced. The change from baseline in VAS score was analyzed as primary endpoint of therapeutic efficacy. The treatment response rate, based on the pre-defined VAS reduction criteria, did not differ significantly between UR-SCIT (78%) and Cluster SCIT (88%) at Month 12 (risk difference: 10%, 95% CI: 29%–9%; *P* = 0.33; Fig. 2D). Comparison of the CSMS-based secondary endpoint yielded comparable response rates between the UR-SCIT and Cluster SCIT groups (90.6% vs 92%, *P* = 0.77). A sensitivity analysis was conducted using Multiple Imputation on the FAS to account for missing data. The FAS analysis of the VAS, RQLQ amd CSMS score at 12 months, based on the Completer Analysis Set, showed a significant improvement in both groups (Supplement Figure 2). The results of VAS were entirely consistent with the primary completer analysis, demonstrating a comparable treatment effect between UR-SCIT and Cluster-SCIT (Mean difference: 0.12, 95% CI: −0.98 to 0.74, *P* = 0.98).

**Fig. 2:**
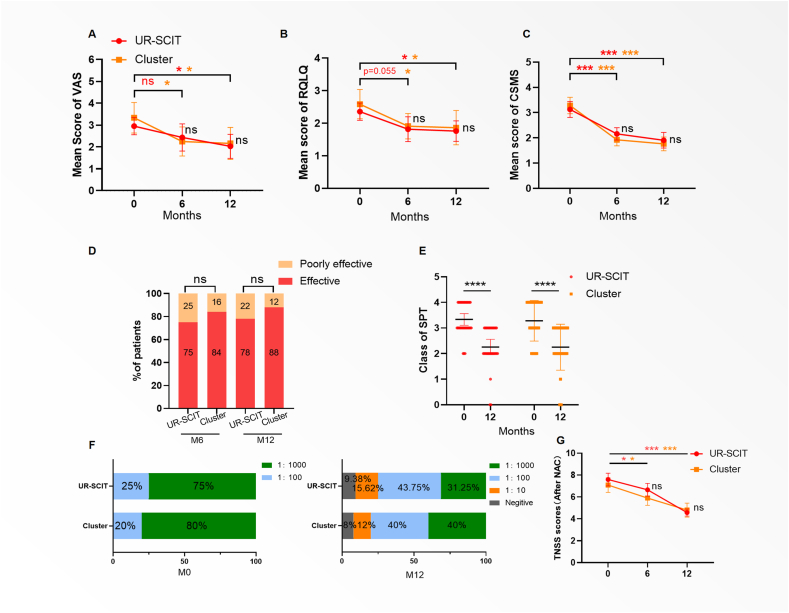
**Changes in the symptom scores following treatment.** (A) Mean VAS scores at M0, M6, and M12. (B) Mean RQLQ scores at M0, M6, and M12. (C) CSMS score.(D) Percentage of effective and ineffective treatments in the 2 groups at M6 and M12. (E) SPT reactivity grades at M0 and M12. (F) Shift in the dose-response relationship of nasal allergen challenge, showing the percentage of reactive patients at different extract concentrations pre- and post-treatment. The rightward shift indicates a decreased sensitivity to the allergen. (G) The associated Total Nasal Symptom Score (TNSS) after challenge. The significant reduction in TNSS at M12 demonstrates improved clinical tolerance. Data are presented as mean with 95% confidence interval. VAS, visual analog scale; RQLQ, Rhinoconjunctivitis Quality of Life Questionnaire; CSMS, combined symptom-medication score; SPT, skin prick test; TNSS, total nasal symptom score; CI, confidence interval. Statistical significance is indicated as ∗∗∗*P* < 0.001, ∗∗*P* < 0.01, and ∗*P* < 0.05

### Allergen sensitivity assessment

2At M12, both groups demonstrated significant reductions in skin prick test (SPT) scores (Fig. 2E). The nasal provocation test also found that the allergen provocation concentration increased in both groups (Fig. 2F). The TNSS also showed a significant decrease at the follow-up point (Fig. 2G).

### Serum immunoglobulins

**Fig. 3:**
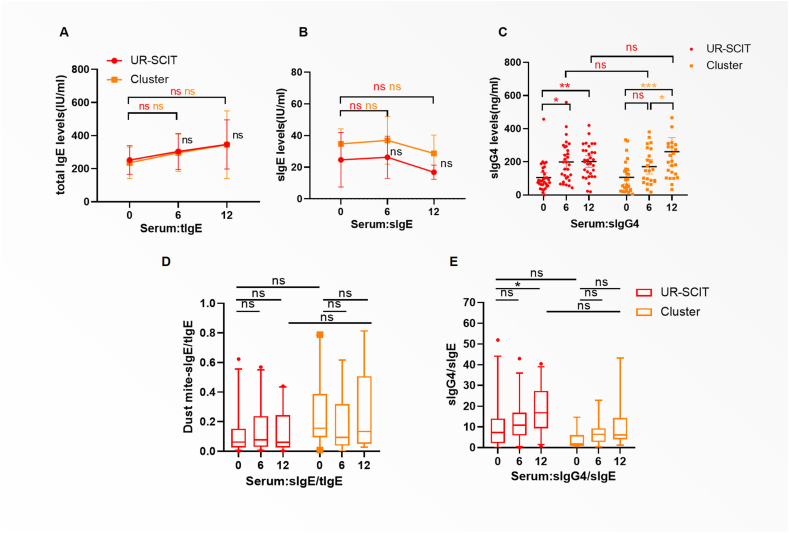
**Changes of immunoglobulins from the baseline.** (A) Total IgE levels at M0, M6, and M12.(B) Specific IgE (sIgE) levels.(C)change from baseline of Dermatophagoides pteronyssinus-specific IgG4 (sIgG4).(D)sIgE/tIgE ratios (E) and sIgG4/sIgE ratios. Data are presented as mean ± 95% confidence interval. Statistical annotations: ∗∗∗*P* < 0.001, ∗∗*P* < 0.01, and ∗*P* < 0.05 for intergroup comparisons (UR-SCIT vs Cluster SCIT).

Total IgE (tIgE) levels increased in both groups during treatment,but no intergroup or intragroup differences were significant (Fig. 3A). sIgE levels transiently rose at M6 but declined by M12, with no statistically significant differences observed either within or between treatment groups (Fig. 3B). Both the Cluster SCIT and UR-SCIT groups exhibited significant increases in allergen-specific IgG4 (sIgG4) levels at 6 and 12 months (Fig. 3C). The ratio of sIgE/tIgE and sIgG4/sIgE showed a numerical increase in both groups after treatment (Fig. 3D–E).

### Immunological parameters

**Fig. 4:**
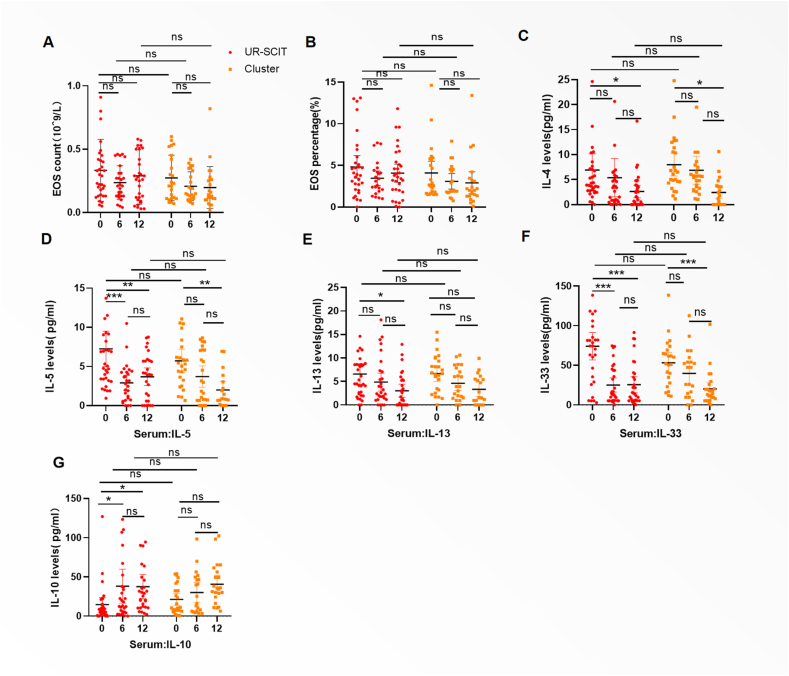
**Levels of cytokines after treatment.** Blood EOS counts (A) and percentages (B) measured at months 0, 6, and 12 (M0, M6, M12). (C–F) Serum concentrations of the type 2 cytokines IL-4 (C), IL-5 (D), IL-13 (E), and the alarmin cytokine IL-33 (F) across the study timepoints. (G) Serum concentrations of IL-10. Data are presented as mean with 95% CI. Statistical comparisons between the Ultra-Rush and Cluster groups at each time point are indicated as ∗∗∗*P* < 0.001, ∗∗*P* < 0.01, and ∗*P* < 0.05. EOS, eosinophils; IL, interleukin; CI, confidence interval

There were no significant changes in the eosinophil count and percentage between 2 groups at M6 and M12 (Fig. 4A–B). At baseline (M0), serum levels of IL-4, IL-5, IL-10, IL-13 and IL-33 showed no significant differences between groups (all *P* > 0.05). Longitudinal assessment from M0 to M12 further revealed progressive reductions in Th2-associated cytokines (IL-4, IL-5, IL-13) and IL-33 were observed at M6 and M12 (Fig. 4C–F). Both immunotherapy regimens induced an increase in IL-10 levels by month 6 (Ultra-rush: Δ = 24.98, *P* < 0.05; Cluster: Δ = 8.70), with a more pronounced numerical increase in the UR-SCIT group compared to the Cluster SCIT group. However, this between-group difference did not reach statistical significance (Mean difference = 8.09, *P* > 0.05). UR-SCIT demonstrated accelerated immunomodulation, with significantly greater suppression of IL-33 compared to Cluster SCIT at the 6-month assessment (Mean difference = −14.15, *P* < 0.05). No significant changes in IL-10 and IL-13 levels were observed in the Cluster group at the 12-month follow-up (Fig. 4E,G).

### Safety comparison between UR-SCIT and cluster SCIT

UR-SCIT patients were observed in hospital for the first 4 days of dose escalation, and Cluster SCIT was treated in outpatient clinic. The incidence of systemic reactions (SRs) was 21.9% (7/32) in the UR-SCIT group and 20% (5/25) in the Cluster group, without significant difference (*P* > 0.05) (Table 3). In the UR-SCIT group, SRs occurred in 12.5% of patients (4/32), while moderate SRs rate were 9.40% (3/32). Among the 800 total injections administered to these 32 patients, 22 injections (2.75%) resulted in mild adverse events, and 10 injections (1.25%) led to moderate SRs. In the Cluster SCIT group, mild systemic adverse reactions occurred in 12.00% of patients (3/25), and moderate SRs were reported in 8% (2/25). 25 patients received 625 injections, among the total injections administered to Cluster SCIT, 19 injections (3.04%) resulted in mild adverse events, and 5 injections (0.8%) led to moderate SRs.

In the UR-SCIT group, systemic adverse events (asthma, wheezing, chest tightness) were resolved after epinephrine, salbutamol or loratadine administration. Local reactions (itching, swelling) were managed with mometasone cream or ice packs. Urticaria is treated with topical corticosteroids combined with oral antihistamines. Notably, 84.4% (27/32) of UR-SCIT adverse events (AEs) occurred within the first 4 days of dose escalation. Only 15.6% AEs were documented during the maintenance period (Table 3). In the Cluster SCIT group, 75% of AEs emerged during the initial phase of treatment.

### Correlation analysis

**Fig. 5:**
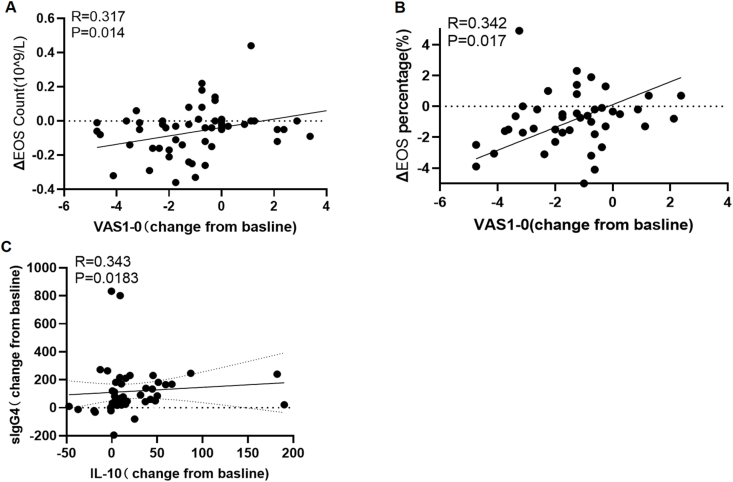
**Correlation analyses of changes in immunologic parameters and clinical symptom scores.** (A) Correlation between the change from baseline in EOS count and the change in VAS score at month 12. (B) Correlation between the change from baseline in EOS percentage and the change in VAS score at month 12. (C) Correlation between the change from baseline in sIgG4 levels and the change from baseline in IL-10 levels at month 12. Data represent the change between baseline (M0) and the 12-month endpoint (M12). Statistical analysis was performed using Pearson correlation, with the correlation coefficient (R) and P-value indicated on each panel. EOS, eosinophils; VAS, Visual Analog Scale; IL, interleukin; sIgG4, specific immunoglobulin G4

In the UR-SCIT and Cluster group,ΔEOS and ΔEOS% was positively correlated with VAS improvement (*R* = 0.317, *P =* 0.014; *R* = 0.342, *P* = 0.017) (Fig. 5A–B). A significant correlation was observed between IL-10 and sIgG4 values (*R* = 0.343, *P* = 0.0183) (Fig. 5C).

## Discussion

This prospective cohort trial demonstrates that UR-SCIT achieves comparable clinical efficacy and safety to Cluster SCIT in house dust mite (HDM)-induced allergic rhinitis (AR), while significantly shortening the dose-escalation phase from 7 weeks to 4 days. Our findings address a critical gap in accelerated immunotherapy for HDM allergy, where prospective evidence remains scarce.^20^^,^^21^ Both regimens significantly improved symptom scores (VAS, RQLQ, CSMS), reduced nasal allergen sensitivity (as shown by SPT and NAC), and suppressed Th2 inflammation, aligning with established accelerated SCIT protocols.^22^, ^23^, ^24^, ^25^

Although UR-SCIT and Cluster SCIT yield comparable clinical outcomes, they appeared to engage distinct immunological pathways. UR-SCIT triggers rapid immunomodulation, characterized by significantly greater suppression of IL-33 and earlier IL-10 surges by Month 6. We hypothesize this intensive allergen exposure could rapidly engage and subsequently exhaust the IL-33/ST2 axis in epithelial and immune cells, leading to the suppression of this pro-inflammatory alarmin observed at M6.^26^, ^27^, ^28^, ^29^, ^30^, ^31^ Concurrently, this robust antigenic challenge appears to preferentially and rapidly expand allergen-specific regulatory T cells (Tregs), driving in an earlier surge of the anti-inflammatory cytokine IL-10.^32^, ^33^, ^34^, ^35^, ^36^ In contrast, the more gradual, weekly escalation in Cluster SCIT provides prolonged yet cumulative stimulation. This protracted antigen exposure appears to favor a slower but steady induction of immune tolerance, primarily driven by a robust humoral response. This is characterized by efficient B-cell activation and maturation, leading to the more pronounced and sustained plasma cell differentiation and allergen-specific IgG4 class-switching that we observed later in the treatment course.^37^, ^38^, ^39^, ^40^ Therefore, the kinetics of antigen delivery fundamentally shapes the immune response: UR-SCIT favors rapid innate immune modulation and early Treg induction, while Cluster SCIT promotes humoral immune adaptation. It should be noted that differing premedication schedules were used during the initial buildup phase. The extended premedication in the UR-SCIT group served as a measure against the elevated risk of SRs posed by its condensed, high-dose escalation protocol—a design critical for patient safety and protocol feasibility. Importantly, all efficacy and immunological assessments (eg, IL-10, IL-33, and sIgG4 levels) were conducted at predefined time points (Months 6 and 12), long after the cessation of premedication. Therefore, we conclude that the immunological differences observed are likely attributable to the distinct allergen exposure dynamics between the 2 regimens, rather than being confounded by transient pharmacological effects of the premedication.

The systemic reaction (SR) rates observed in both UR-SCIT (21.9%) and Cluster SCIT (20%) cohorts lign with prior accelerated immunotherapy studies (15–25% range).^19^^,^^41^ While some earlier studies support the feasibility of accelerated immunotherapy protocols, their safety assessments vary considerably. Casanovas et al achieved ultra-rush immunotherapy in 1068 pollen/HDM-allergic patients without premedication, reporting only grade 1–2 self-resolving reactions.^42^ Conversely, retrospective analyses of UR-SCIT in mixed cohorts (AR with asthma/AD) documented SAR rates of 11.5% (including 1 laryngeal edema case),^43^ whereas Nazila Ariaee et al demonstrated ultra-rush immunotherapy's safety with antihistamine premedication in perennial AR.^44^ Our prospective design, which involved real-time documentation of adverse events in a homogeneous AR population (excluding comorbid asthma or atopic dermatitis), may account for the relatively higher SR incidence compared to these retrospective analyses.^45^ Reassuringly, no life-threatening reactions (Grade ≥3) occurred, validating the efficacy of our standardized premedication protocol—oral corticosteroids combined with second-generation antihistamines—in suppressing early-phase mast cell activation. The standardized premedication during buildup was essential for mitigating SR risks.

The favorable safety and efficacy of UR-SCIT observed in our HDM-AR cohort warrants comparison with the established use of ultra-rush protocols for other allergens. For instance, ultra-rush regimens have demonstrated an excellent safety profile with only infrequent and mild anaphylactic reactions in bee and vespid venom immunotherapy.^22^ Similarly, UR-SCIT has been effectively employed for pollen allergies to reach maintenance dose before the pollen season.^46^^,^^47^ Research on HDM immunotherapy has historically emphasized conventional or Cluster AIT, with the limited existing UR-SCIT data primarily coming from retrospective analyses or mixed allergen cohorts that often included patients with asthma.^48^ Our prospective cohort study provides a direct, head-to-head comparison of UR-SCIT and the Cluster SCIT in a homogeneous HDM-AR population, we demonstrate that the significant logistical advantage of a 4-day buildup phase is achievable for HDM allergy without compromising safety. This firmly positions UR-SCIT as a viable and efficient new option within the AIT protocals for this prevalent perennial allergy.

From a clinical implementation perspective, UR-SCIT addresses a key barrier to adherence by drastically shortening the initial buildup phase. In our cohort, the 12-month adherence rate for UR-SCIT reached 80%, surpassing rates typically reported for conventional SCIT. While Ji Ho Lee's study reported higher treatment adherence in Cluster SCIT compared to UR-SCIT,^49^ our trial demonstrated comparable adherence rates between the 2 groups (Fig. 5A). This equivalence may be attributed to the optimized premedication regimen (eg, corticosteroids and H1-antagonists) administered during the initial dose-escalation phase, which effectively mitigated the early-phase adverse reactions and improved adherence in the UR-SCIT cohort.

This single-center trial has some limitations. First, the non-randomized allocation of patients based on their preference introduces a potential for selection bias. Although we attempted to mitigate this by comparing baseline characteristics, which showed no significant differences, residual confounding factors cannot be ruled out. Second, mechanistic insights were constrained to serum biomarkers—tissue-level changes (eg, nasal eosinophils and IL-10+ Treg infiltration) remain uncharacterized. Third, the one-year follow-up duration limits our ability to assess the long-term durability of clinical efficacy and immunological tolerance. Future studies with extended follow-up (3–5 years) and integrated advanced techniques (eg, single-cell RNA sequencing) are warranted to determine whether the early immunological changes observed in UR-SCIT translate into sustained therapeutic benefit.

## Conclusion

UR-SCIT serves as a safe, efficient alternative to Cluster SCIT, achieving comparable efficacy through rapid IL-33 suppression, early IL-10 induction and sIgG4 class switching. Our premedication protocol successfully mitigated SR risks inherent to accelerated schedules, enabling a 4-day buildup phase without compromising safety. Our results support the adoption of UR-SCIT in clinical practice, especially for patients prone to non-adherence. Future studies with extended follow-up are warranted to verify the long-term durability of the observed immunological and clinical effects. Furthermore, elucidating the precise mechanisms of action will pave the way for its broader clinical applications.

## Availability of data and materials

Not applicable

## CRediT authorship contribution statement

**Yishan XIONG:** Conceptualization, Formal analysis, Investigation, Project administration, Validation, Visualization, Writing. **Wenqi LUO**:Investigation, Validation, Visualization**. Hao PENG**: Conceptualization, Formal analysis, Investigation, Project administration. **Li SHEN**:Validation, Visualization. **Zhiqun HUANG**: Validation, Visualization. **Jieqing YU:** Validation, Visualization. **Jing YE**: Conceptualization, Formal analysis, Funding acquisition, Investigation, Project administration, Supervision, Validation, Visualization, Writing—review and editing.

## Ethics approval and consent to participate

Trial registration: Chinese Clinical Trial Registry (ChiCTR2200061409); Registered June 23, 2022 (prospectively registered). The study was approved by the Medical Research Ethics Committee of the First Affiliated Hospital of Nanchang University (Approval No.:ⅡT 2022–32) as per the Declaration of Helsinki. Informed written consent was obtained from all patients. All participants provided written consent for the publication of de-identified aggregate data. The manuscript contains no individually identifiable patient data, images, or videos requiring additional consent.

## Author consent for publication

All authors reviewed the final version of the manuscript and approved publication in this journal.

## Authors’ statement on the use of generative AI

We confirm that no generative artificial intelligence (AI) or AI-assisted technologies were employed during the Writing of this work.

## Funding

This study was supported by the Research Cultivation Project of Jiangxi Institute of Nutrition and 10.13039/100018696Health Management (No. 2022-PYXM-05), the 10.13039/501100001809National Natural Science Foundation of China (Grant Nos. 81860182 and 82360219), and the Clinical Research Cultivation Program of The First Affiliated Hospital of 10.13039/501100004637Nanchang University (No. YFYLCYJPY202304).

## Declaration of competing interest

The authors declare that they have no competing interests that could have appeared influence the work in this paper.

## Figures and Tables

**figs1:**
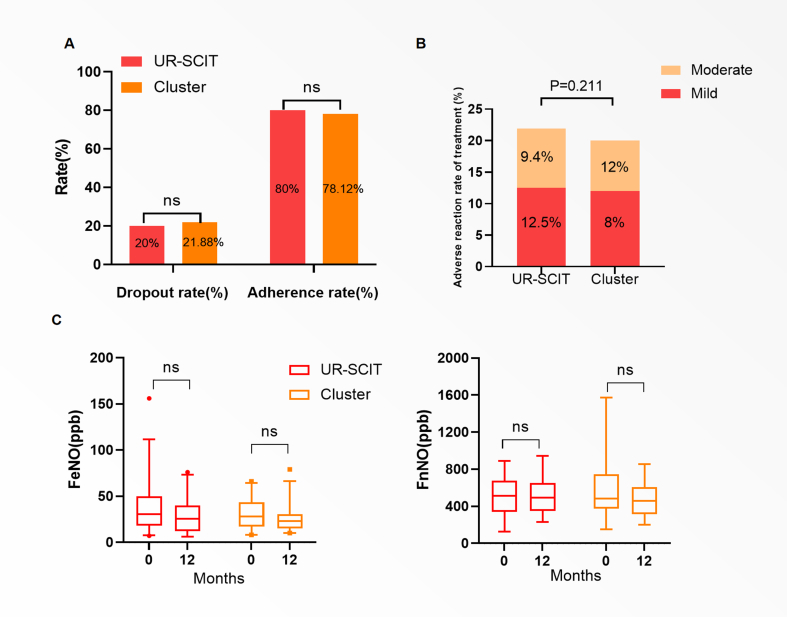


**figs2:**
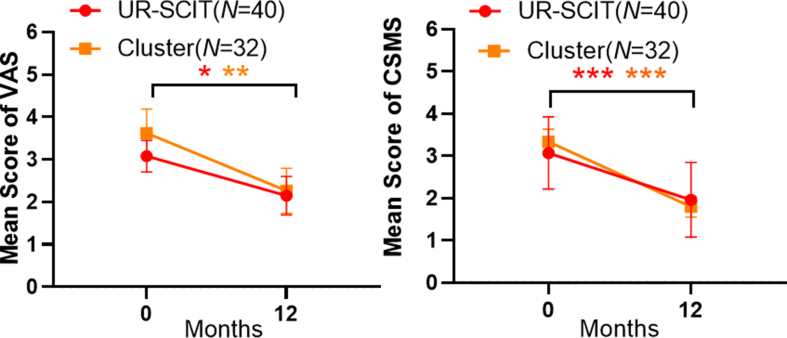


## References

[1] Lee H.Y., Lee S.M., Kang S.Y. (2023). KAAACI guidelines for allergen immunotherapy. Allergy Asthma Immunol Res.

[2] Bousquet J., Anto J.M., Bachert C. (2020). Allergic rhinitis. Nat Rev Dis Primers.

[3] Aarestrup F.M., Taketomi E.A., Santos Galvão C.E. (2022). Good clinical practice recommendations in allergen immunotherapy: position paper of the Brazilian association of allergy and immunology - ASBAI. World Allergy Organ J.

[4] Paoletti G., Nappi E., Bragato M.C. (2024). Allergen immunotherapy in Italy: how, when, and why-A real-world study conducted through a patient association. World Allergy Organ J.

[5] Caruso C., Colantuono S., Tolusso B. (2023). Effects of house dust mite subcutaneous immunotherapy in real-life. Immunological and clinical biomarkers and economic impact analysis. World Allergy Organ J.

[6] Penagos M., Durham S.R. (2022). Allergen immunotherapy for long-term tolerance and prevention. J Allergy Clin Immunol.

[7] Creticos P.S., Gunaydin F.E., Nolte H., Damask C., Durham S.R. (2024). Allergen immunotherapy: the evidence supporting the efficacy and safety of subcutaneous immunotherapy and sublingual forms of immunotherapy for allergic rhinitis/conjunctivitis and asthma. J Allergy Clin Immunol Pract.

[8] Jutel M., Klimek L., Richter H., Brüggenjürgen B., Vogelberg C. (2024). House dust mite SCIT reduces asthma risk and significantly improves long-term rhinitis and asthma control-A RWE study. Allergy.

[9] Durham S.R., Shamji M.H. (2023). Allergen immunotherapy: past, present and future. Nat Rev Immunol.

[10] Rodríguez-Domínguez A., Berings M., Rohrbach A. (2020). Molecular profiling of allergen-specific antibody responses may enhance success of specific immunotherapy. J Allergy Clin Immunol.

[11] Głobińska A., Boonpiyathad T., Satitsuksanoa P. (2018). Mechanisms of allergen-specific immunotherapy: diverse mechanisms of immune tolerance to allergens. Ann Allergy Asthma Immunol.

[12] Huang Y., Wang C., Wang X., Zhang L., Lou H. (2019). Efficacy and safety of subcutaneous immunotherapy with house dust mite for allergic rhinitis: a meta-analysis of randomized controlled trials. Allergy.

[13] Huang J., Zhang W., Xiang R. (2023). The early-phase transcriptome and the clinical efficacy analysis in three modes of subcutaneous immunotherapy for allergic rhinitis. World Allergy Organ J.

[14] Zhang P., Bian S., Wang X. (2022). A real-world retrospective study of safety, efficacy, compliance and cost of combination treatment with rush immunotherapy plus one dose of pretreatment anti-IgE in Chinese children with respiratory allergies. Front Immunol.

[15] Huang J.Y., Zhang W., Xiang R., Deng Y.Q., Tao Z.Z., Xu Y. (2023). Short-term efficacy and safety observation of standardized mite allergen extract rush subcutaneous immunotherapy for allergic rhinitis: a prospective study. Zhonghua Er Bi Yan Hou. Tou Jing Wai Ke Za Zhi.

[16] Uriarte S., Sastre J. (2018). Safety of an ultrarush (4 hours) subcutaneous immunotherapy schedule with cat and dog extracts using an infusion pump. J Investig Allergol Clin Immunol.

[17] Temiño V.M., Wu P., Konig J. (2013). Safety of multiple aeroallergen rush immunotherapy using a modified schedule. Allergy Asthma Proc.

[18] Bousquet J., Khaltaev N., Cruz A.A. (2008). Allergic Rhinitis and its impact on Asthma (ARIA) 2008 update (in collaboration with the World Health Organization, GA(2)LEN and AllerGen). Allergy.

[19] Turner P.J., Ansotegui I.J., Campbell D.E. (2024). Updated grading system for systemic allergic reactions: joint statement of the world allergy organization anaphylaxis committee and allergen immunotherapy committee. World Allergy Organ J.

[20] Balaji R., Parasuramalu B.G., Chandregowda B.V. (2014). Gangaboraiah. Safety, tolerability and clinical efficacy of ultra-rush sublingual immunotherapy among patients suffering from allergic rhinitis. Allergol Immunopathol.

[21] Wang L., Wang C., Lou H., Zhang L. (2021). Antihistamine premedication improves safety and efficacy of allergen immunotherapy. Ann Allergy Asthma Immunol.

[22] Stock R., Fischer T., Aßmus K. (2020). Safety and tolerability of venom immunotherapy: evaluation of 581 rush- and ultra-rush induction protocols (safety of rush and ultra-rush venom immunotherapy). World Allergy Organ J.

[23] Bergmann K.C., Demoly P., Worm M. (2014). Efficacy and safety of sublingual tablets of house dust mite allergen extracts in adults with allergic rhinitis. J Allergy Clin Immunol.

[24] Kim M.E., Kim J.E., Sung J.M., Lee J.W., Choi G.S., Nahm D.H. (2011). Safety of accelerated schedules of subcutaneous allergen immunotherapy with house dust mite extract in patients with atopic dermatitis. J Kor Med Sci.

[25] Cayrol C., Girard J.P. (2014). IL-33: an alarmin cytokine with crucial roles in innate immunity, inflammation and allergy. Curr Opin Immunol.

[26] Peng Y.Q., Chen D.H., Xu Z.B. (2021). IL-33 receptor expression on myeloid and plasmacytoid dendritic cells after allergen challenge in patients with allergic rhinitis. Int Immunopharmacol.

[27] Shamji M.H., Kappen J.H., Akdis M. (2017). Biomarkers for monitoring clinical efficacy of allergen immunotherapy for allergic rhinoconjunctivitis and allergic asthma: an EAACI position paper. Allergy.

[28] Layhadi J.A., Lalioti A., Palmer E., van Zelm M.C., Wambre E., Shamji M.H. (2024). Mechanisms and predictive biomarkers of allergen immunotherapy in the clinic. J Allergy Clin Immunol Pract.

[29] Drazdauskaitė G., Layhadi J.A., Shamji M.H. (2020). Mechanisms of allergen immunotherapy in allergic rhinitis. Curr Allergy Asthma Rep.

[30] Liew F.Y., Girard J.P., Turnquist H.R. (2016). Interleukin-33 in health and disease. Nat Rev Immunol.

[31] Zemelka-Wiacek M., Agache I., Akdis C.A. (2024). Hot topics in allergen immunotherapy, 2023: current status and future perspective. Allergy.

[32] Yang L., Yang Y., Xu Q. (2022). Specific IgE and IgG4 profiles of house dust mite components in allergen-specific immunotherapy. Front Immunol.

[33] Feng M., Zeng X., Su Q. (2020). Allergen immunotherapy-induced immunoglobulin G4 reduces Basophil activation in house dust mite-allergic asthma patients. Front Cell Dev Biol.

[34] Akdis M., Akdis C.A. (2014). Mechanisms of allergen-specific immunotherapy: multiple suppressor factors at work in immune tolerance to allergens. J Allergy Clin Immunol.

[35] Berings M., Karaaslan C., Altunbulakli C. (2017). Advances and highlights in allergen immunotherapy: on the way to sustained clinical and immunologic tolerance. J Allergy Clin Immunol.

[36] Fajt M.L., Rosenberg S.L., Yecies E., Traister R.S., Petrov A.A. (2017). A 10-year experience of a novel and safe modified environmental rush immunotherapy protocol. Allergy Asthma Proc.

[37] Boonpiyathad T., Satitsuksanoa P., Akdis M., Akdis C.A. (2019). Il-10 producing T and B cells in allergy. Semin Immunol.

[38] Eggel A., Pennington L.F., Jardetzky T.S. (2024). Therapeutic monoclonal antibodies in allergy: targeting IgE, cytokine, and alarmin pathways. Immunol Rev.

[39] Breiteneder H., Peng Y.Q., Agache I. (2020). Biomarkers for diagnosis and prediction of therapy responses in allergic diseases and asthma. Allergy.

[40] Shamji M.H., Valenta R., Jardetzky T. (2021). The role of allergen-specific IgE, IgG and IgA in allergic disease. Allergy.

[41] Gu T., Zhang W., Tan L. (2025). Intratonsillar immunotherapy: a convenient and effective alternative to subcutaneous immunotherapy for allergic rhinitis. Research.

[42] Casanovas M., Martín R., Jiménez C., Caballero R., Fernández-Caldas E. (2006). Safety of an ultra-rush immunotherapy build-up schedule with therapeutic vaccines containing depigmented and polymerized allergen extracts. Int Arch Allergy Immunol.

[43] Lee S.H., Kim M.E., Shin Y.S., Ye Y.M., Park H.S., Nahm D.H. (2019). Safety of ultra-rush schedule of subcutaneous allergen immunotherapy with house dust mite extract conducted in an outpatient clinic in patients with atopic dermatitis and allergic rhinitis. Allergy Asthma Immunol Res.

[44] Ariaee N., Fouladvand A., Mohammadi M. (2024). A modified schedule of multiple aeroallergen ultra-rush immunotherapy in perennial allergic rhinitis: safety, efficacy, and T lymphocyte cell population studies. Allergol Immunopathol.

[45] Cardona R., Lopez E., Beltrán J., Sánchez J. (2014). Safety of immunotherapy in patients with rhinitis, asthma or atopic dermatitis using an ultra-rush buildup. A retrospective study. Allergol Immunopathol.

[46] Mösges R., Graute V., Christ H., Sieber H.J., Wahn U., Niggemann B. (2010). Safety of ultra-rush titration of sublingual immunotherapy in asthmatic children with tree-pollen allergy. Pediatr Allergy Immunol.

[47] Pfaar O., Urry Z., Robinson D.S. (2012). A randomized placebo-controlled trial of rush preseasonal depigmented polymerized grass pollen immunotherapy. Allergy.

[48] Seidenberg J., Pajno G.B., Bauer C.P., La Grutta S., Sieber J. (2009). Safety and tolerability of seasonal ultra-rush, high-dose sublingual-swallow immunotherapy in allergic rhinitis to grass and tree pollens: an observational study in 193 children and adolescents. J Investig Allergol Clin Immunol.

[49] Lee J.H., Lee S.H., Ban G.Y. (2019). Factors associated with adherence to allergen specific subcutaneous immunotherapy. Yonsei Med J.

