# Supplementary material for: A prospective cohort study of ultra-rush subcutaneous immunotherapy in dust mite-induced allergic rhinitis
Source: World Allergy Organ J. 2026 Feb 6;19(2):101334. doi: 10.1016/j.waojou.2026.101334 (PMC12907656; doi:10.1016/j.waojou.2026.101334)

**Supplementary Table 1. Characteristics and management of systemic reactions (SRs) during treatment.**

| Patient | Gender(M/F) | Age(yr) | Type of SR | Severity grade of SR | Allergen dose that induced SR(SQ-U) | Treatment for SR | Administration of maxiumum allergen dose during UR-SCIT | Dose reduction because of SR | Treatment cessation because of SR |
| --- | --- | --- | --- | --- | --- | --- | --- | --- | --- |
| UR-SCIT |  |  |  |  |  |  |  |  |  |
| 7 | F | 33 | Chest congestion,pruritus | 2 | 10,000 | B, C | Yes | No | No |
| 11 | F | 24 | Generalized pruritus of skin,Urticaria | 2 | 50,000 | A, B | Yes | No | No |
| 18 | M | 19 | Chest congestion,localized swelling of skin | 2 | 40,000 | B, C | Yes | No | No |
| 22 | M | 15 | Chest congestion,localized swelling of skin | 2 | 50,000 | B, C | Yes | No | No |
| 29 | F | 37 | Urticaria,,rhinitis | 1 | 40,000 | A, B | Yes | No | No |
| 31 | M | 16 | Generalized pruritus of skin,nausea and headache | 2 | 100,000 | A, B | Yes | No | No |
| 32 | F | 24 | Urticaria,conjunctival erythema | 1 | 20,000 | A, B | Yes | No | No |
| Cluster SCIT |  |  |  |  |  |  |  |  |  |
| 40 | F | 26 | Urticaria | 1 | 100,000 | NO | Yes | No | No |
| 42 | M | 36 | chest congestion,nausea,rhinitis | 2 | 40,000 | A, C | Yes | No | No |
| 47 | F | 51 | Urticaria,conjunctival erythema | 1 | 60,000 | A | Yes | No | No |
| 52 | M | 33 | Urticaria,localized swelling of skin | 1 | 20,000 | A | Yes | No | No |
| 54 | F | 56 | Urticaria,rhinitis | 1 | 40,000 | A, B | Yes | No | No |
| Severity was graded as 1 (Mild) or 2 (Moderate). Treatment: A, Antihistamines; B, Glucocorticoids; C, β2-agonist. | | | | | | | | | |

**Supplementary Table 2. Changes in Patient-Reported Outcome Measures from Baseline to Month 12 (M12) in the Full Analysis Set (FAS) and Completer Analysis Set (CAS). Data are presented as Mean (95% Confidence Interval). Negative values for the VAS，CSMS and RQLQ indicate improvement. FAS, Full Analysis Set; CAS, Completer Analysis Set; CSMS, Combined Symptom and Medication Score; RQLQ, Rhinoconjunctivitis Quality of Life Questionnaire; VAS, Visual Analog Scale.**

| **Outcome Measure** | **Comparison** | **Time Point** | **FAS (*N*= 72), Mean(95%Cl)** | **CAS (*N*= 57), Mean(95%Cl)** |
| --- | --- | --- | --- | --- |
| VAS | UR-SCIT vs. Cluster SCIT | M0-M12 | -0.87 (-1.75 to -0.25) vs. -0.37 (-2.25 to -0.12) | -0.93 (-1.75 to -0.25) vs. -0.76 (-2.15 to -0.64) |
| CSMS | UR-SCIT vs. Cluster SCIT | M0-M12 | -1.15 (-2.0 to -1.0) vs. -1.52 (-2.0 to -1.0) | -1.11 (-1.14 to -1.0) vs. -1.63(-2.0 to -1.0) |
| RQLQ | UR-SCIT vs. Cluster SCIT | M0-M12 | -0.58 (-0.88 to -0.32) vs. -0.46 (-1.03 to -0.25) | -0.52 (-0.72 to -0.38) vs. -0.43 (-1.10 to -0.25) |

**Supplementary Figures.**

**Figure 1.Treatment adherence and adverse event profile.** (A) Bar graph showing the adherence rate (percentage of patients completing the 12-month protocol) and the dropout rate for the Ultru-rush and Cluster SCIT groups. (B) Stacked bar graph displaying the severity distribution of systemic adverse reactions among the affected patients in each group, categorized as mild or moderate. The P-value for the comparison of adherence rates between groups is shown. Statistical annotation: ns, not significant. (C) Changes of FeNO and FnNO at M12 .


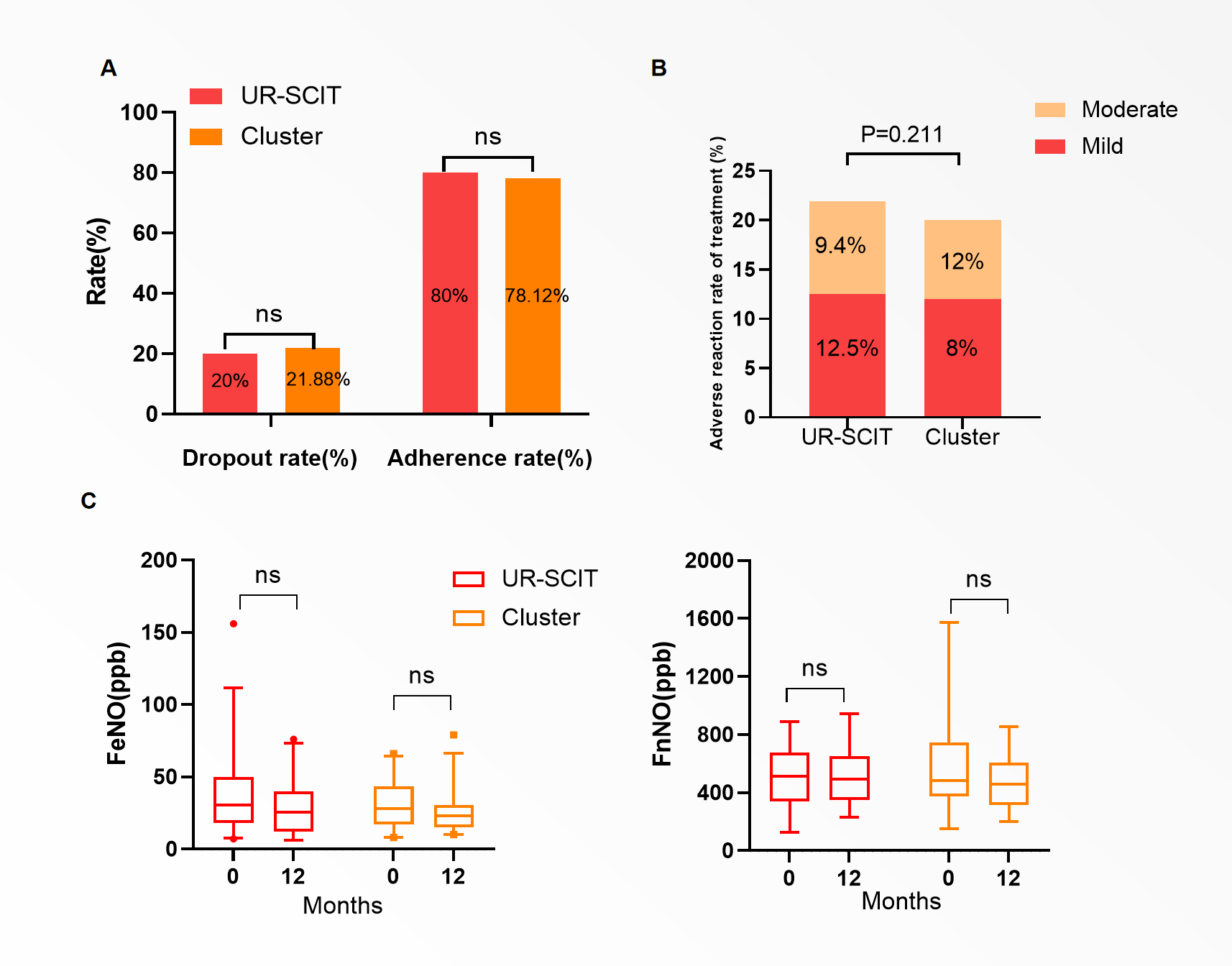


**Figure 2. Evolution of the VAS and CSMS Score over time.** (A-B) The line graph depicts the mean VAS and CSMS for the Ultru-rush SCIT (*N*=40) and Cluster SCIT (*N*=32) groups from baseline (M0) to the 12-month endpoint (M12), based on the Full Analysis Set (FAS). The error bars represent the mean with 95% CI at each time point. Statistical significance between groups at each time point is indicated as ****P* < 0.001, ***P* < 0.01, and **P* < 0.05. CI, confidence interval.


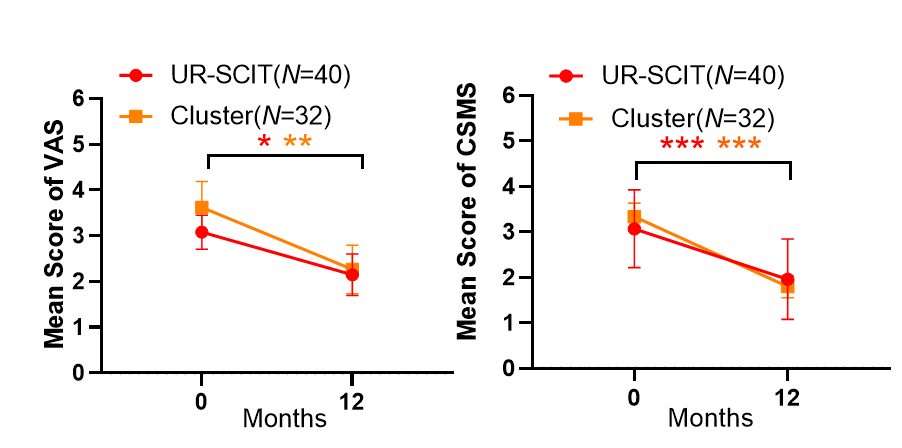

Supplement: Multimedia component 1 [file mmc1.docx]
